# Diagnostic Accuracy of Sputum and Bronchoscopy-Guided Cytology Compared With Bronchial Biopsy in Pulmonary Lesions

**DOI:** 10.7759/cureus.108269

**Published:** 2026-05-04

**Authors:** Suganthi P, Twinkle Dhamecha, Nilam Patel, Vinod Kumar

**Affiliations:** 1 Pathology, Government Pudukkottai Medical College and Hospital, Pudukkottai, IND; 2 Microbiology, WePath Laboratory, Vadodara, IND; 3 Pathology, Dr. N.D. Desai Faculty of Medical Science and Research, Dharmsinh Desai University, Nadiad, IND; 4 Pathology, Government Medical College, Datia, IND

**Keywords:** bronchial biopsy, bronchoalveolar lavage, diagnostic accuracy, pulmonary malignancy, pulmonary pathology, sputum cytology, transbronchial needle aspiration

## Abstract

Background: Bronchial biopsy remains the reference standard for diagnosing pulmonary pathology; however, minimally invasive cytological techniques are increasingly used in clinical practice. This study aimed to determine the diagnostic accuracy of sputum cytology, bronchoalveolar lavage (BAL) cytology, transbronchial needle aspiration (TBNA), and bronchial brush cytology in detecting malignant pulmonary lesions using bronchial biopsy as the reference standard, and to compare their relative diagnostic performance.

Methods: A prospective observational study was conducted at Government Medical College, Datia, from March to September 2025. A total of 120 adult patients with clinical and radiological suspicion of pulmonary pathology underwent sputum cytology and bronchoscopy-guided sampling (BAL, TBNA, brush cytology), followed by bronchial biopsy. Histopathology served as the reference standard. Diagnostic performance parameters were calculated. Suspicious cytology results were considered positive for malignancy for sensitivity analysis.

Results: Of 120 patients, 77 (64.2%) had malignant lesions on biopsy. TBNA cytology demonstrated the highest sensitivity (92.2%) and excellent diagnostic accuracy (area under the curve (AUC) 0.969), followed by brush cytology (sensitivity 85.7%, AUC 0.974). BAL cytology showed moderate sensitivity (71.4%, AUC 0.887), while sputum cytology had lower sensitivity (49.4%, AUC 0.808). All modalities showed statistically significant associations with histopathology (p < 0.001). TBNA and brush cytology exhibited superior concordance with biopsy findings compared with BAL and sputum cytology.

Conclusion: Bronchoscopy-guided cytological techniques, particularly TBNA and brush cytology, demonstrate high diagnostic accuracy and strong concordance with bronchial biopsy findings. These modalities serve as valuable complementary diagnostic tools in selected clinical settings but do not replace histopathological confirmation.

## Introduction

Pulmonary pathology encompasses malignant, infectious, and interstitial lung diseases. Timely, accurate diagnosis is critical for effective management. Bronchoscopy plays a key role in evaluating suspected lung disease, particularly cancer, with histopathological confirmation guiding staging and treatment. A large study of 5,279 bronchoscopies found that the diagnostic effectiveness of bronchoscopic biopsy depends on tumor location and histological subtype, underscoring the importance of selecting appropriate sampling methods [1].

Despite technological advances, the diagnostic yield of guided bronchoscopy for peripheral pulmonary lesions remains approximately 70% [2]. Yield is influenced by lesion size, bronchus sign, and malignancy prevalence [2]. Inconsistent definitions of diagnostic yield also affect reported effectiveness, highlighting the need for standardized endpoints [3]. These factors raise important questions about the comparative value of cytological and histological techniques.

Rapid on-site evaluation (ROSE) of transbronchial needle aspiration (TBNA) samples achieves high sensitivity (89.6%) and specificity (95.9%) when performed by trained pulmonologists. Cytology provides reliable diagnostic adequacy, even in resource-limited settings [4]. Endobronchial ultrasound-guided TBNA (EBUS-TBNA) cytological specimens suffice for diagnosis and molecular profiling in non-small cell lung cancer (NSCLC), matching biopsy samples in genetic and immunohistochemical analyses [5]. These findings underscore the expanding role of minimally invasive cytological approaches in oncologic diagnostics.

CT-guided transthoracic biopsy is most accurate but risks more complications. Newer bronchoscopic methods, like robotic-assisted bronchoscopy, are as accurate and safer [6]. In infectious pulmonary disease, bronchoalveolar lavage (BAL) matches bronchial washings and sputum moderately for certain pathogens, so less invasive samples may sometimes suffice [7]. In pediatric pneumonia, sputum and BAL fluid often harbour the same pathogens, supporting their interchangeable use under certain conditions [8].

However, growing evidence supports the use of sputum cytology and bronchoscopy-guided techniques (including BAL fluid analysis, TBNA, and bronchial brushings) for diagnosing malignant and non-malignant pulmonary conditions. These techniques vary in diagnostic yield, adequacy, and agreement, so we must systematically compare them to bronchial biopsy. Understanding the diagnostic performance of these modalities is crucial to optimize minimally invasive diagnostic pathways, particularly in resource-limited settings. Therefore, the present study aimed to determine the diagnostic accuracy of sputum cytology, BAL cytology, TBNA, and bronchial brush cytology for detecting malignant pulmonary lesions using bronchial biopsy as the reference standard, and to compare their relative diagnostic performance across modalities.

## Materials and methods

Study design

This prospective observational study was conducted to evaluate the diagnostic accuracy of sputum cytology and bronchoscopy-guided cytological techniques (BAL, TBNA, and bronchial brush cytology) using bronchial biopsy as the reference standard.

Study setting and duration

The study was carried out in the Department of Pathology, Government Medical College, Datia, in collaboration with the Department of Pulmonary Medicine, Government Medical College, Datia. All cytological and histopathological processing and reporting were performed in the Department of Pathology using standardized laboratory protocols. The study was conducted over a period of six months, from March 2025 to September 2025.

Study population

The study population comprised 120 consecutive adult patients presenting with clinical symptoms and radiological findings suggestive of pulmonary pathology at Government Medical College, Datia, during the study period. Clinical features included persistent cough, hemoptysis, dyspnea, unexplained weight loss, chest pain, or prolonged fever. Radiological suspicion was based on findings such as lung mass, pulmonary nodule, non-resolving consolidation, cavitary lesion, or mediastinal/hilar lymphadenopathy on chest radiograph or CT. All enrolled patients were planned for diagnostic bronchoscopy as part of their routine clinical evaluation.

Inclusion criteria

Patients were included if they were aged 18 years or older, had clinical and radiological suspicion of pulmonary pathology requiring bronchoscopic evaluation, and provided written informed consent for participation. Only those patients in whom sputum cytology, bronchoscopy-guided cytological sampling (BAL, TBNA, and bronchial brush), and bronchial biopsy could be performed during the same diagnostic workup were included to allow uniform comparison.

Exclusion criteria

Patients were excluded if they were medically unfit for bronchoscopy due to severe hypoxemia (peripheral oxygen saturation (SpO₂) <90% on room air), uncontrolled coagulopathy (international normalized ratio (INR) >1.5 or platelet count <50,000/mm³), significant cardiovascular instability, or other contraindications to the procedure. Patients with a previously established histopathological diagnosis of pulmonary disease, those unwilling to provide consent, and cases with inadequate or poorly preserved cytological or biopsy specimens were also excluded from the analysis.

Lesion characteristics

Where available, lesion characteristics such as anatomical location (central vs peripheral), radiological morphology, and size were recorded. However, detailed stratified subgroup analysis based on these variables was not performed and is acknowledged as a limitation.

Sputum cytology

All patients were instructed to provide three early-morning deep cough sputum samples on consecutive days prior to bronchoscopy. Samples with excessive salivary contamination were rejected. Specimens were processed using cytocentrifugation at 1,500 rpm for 10 minutes. Smears were prepared from the sediment and fixed in 95% ethanol. Staining was performed using Papanicolaou and May-Grünwald-Giemsa (MGG) stains (Merck KGaA, Germany). Cytological findings were categorized as malignant, suspicious for malignancy, specific benign pathology (e.g., granulomatous inflammation), nonspecific inflammatory/negative for malignancy, or inadequate.

Bronchoscopy procedure and sample collection

Flexible fiberoptic bronchoscopy was performed using an Olympus BF-1T180 bronchoscope (Olympus Corporation, Japan) under local anesthesia with 2% lignocaine, with or without conscious sedation, under continuous monitoring using a multiparameter monitor (BPL Medical Technologies, India). Parameters monitored included pulse rate, blood pressure, oxygen saturation, and electrocardiogram. Lesions were localized based on prior CT imaging. Samples were collected sequentially to maintain uniformity.

BAL was performed by wedging the bronchoscope into the affected segment and instilling 100 mL of sterile normal saline (Baxter International Inc., USA) in 20 mL aliquots. The fluid was gently aspirated after each aliquot and collected in sterile containers. BAL samples were centrifuged at 1500 rpm for 10 minutes using a laboratory centrifuge (Remi Elektrotechnik Ltd., India), and smears were prepared from the sediment and stained with Papanicolaou and MGG stains.

Bronchial brush cytology was obtained using a sterile cytology brush (ConMed Corporation, USA) introduced through the working channel and applied directly to visible lesions or suspicious mucosa. Five to ten brushing strokes were performed. The brush was rolled onto clean glass slides, immediately fixed in 95% ethanol (Merck KGaA, Germany), and processed for staining.

TBNA was performed using a 21-22-gauge aspiration needle (Cook Medical, USA) introduced through the bronchoscope into the target lesion or an enlarged lymph node. Negative pressure was applied using a 10 mL syringe (Hindustan Syringes & Medical Devices Ltd., India), and aspirated material was expelled onto glass slides. Air-dried smears were prepared for MGG staining and alcohol-fixed smears for Papanicolaou staining. TBNA was performed from endobronchial lesions or from accessible lymph nodes, as identified by bronchoscopic findings. A minimum of two-three passes were attempted per lesion where feasible. All bronchoscopic procedures were performed by experienced pulmonologists. ROSE was not routinely utilized.

Bronchial biopsy was subsequently performed from the same lesion site using endobronchial forceps (Olympus Corporation, Japan). A minimum of three to five tissue fragments were obtained per patient. Specimens were fixed in 10% neutral buffered formalin (Merck KGaA, Germany) for 24 hours, processed routinely, embedded in paraffin, and sectioned at 3-4 µm thickness using a microtome (Leica Biosystems, Germany). Sections were stained with hematoxylin and eosin (Merck KGaA, Germany). Special stains and immunohistochemistry were performed when indicated. Histopathological diagnosis from bronchial biopsy served as the gold standard. 

Cytological findings categorized as “suspicious for malignancy” were considered positive for diagnostic accuracy calculations. Inadequate samples were excluded from the sensitivity and specificity analysis but reported separately.

All cytological and histopathological evaluations were performed by experienced pathologists using standardized criteria. Although formal interobserver variability analysis was not conducted, internal quality checks and consensus discussions were undertaken for challenging cases.

Blinding

Cytological specimens were evaluated independently by pathologists who were blinded to the histopathological diagnosis. Histopathological evaluation was performed separately to minimize observer bias.

Outcome measures

The primary outcome was the correlation between cytological findings (sputum, BAL, TBNA, and bronchial brush cytology) and bronchial biopsy histopathology. Secondary outcomes included calculation of sensitivity, specificity, positive predictive value (PPV), negative predictive value (NPV), diagnostic accuracy, and concordance rates for each cytological modality.

Statistical analysis

Data were entered into Microsoft Excel and analysed using Jamovi version 2.6.44. Categorical variables were expressed as frequencies and percentages. Diagnostic performance parameters were calculated using standard 2×2 contingency tables with bronchial biopsy as the reference standard. Sensitivity, specificity, PPV, NPV, and overall diagnostic accuracy were computed for each cytological modality. Agreement between cytological findings and histopathology was assessed using Cohen’s kappa coefficient. A p-value <0.05 was considered statistically significant.

Ethical considerations

The study was approved by the institutional ethics committee of Government Medical College, Datia. Written informed consent was obtained from all participants prior to enrollment. Confidentiality and anonymity of patient information were maintained throughout the study.

## Results

A total of 120 participants were included in the study, and complete data were available for all variables. The mean age of the study population was 53.5 ± 14.5 years, with a median of 53 years and a range of 30 to 79 years, indicating that the majority of patients were middle- to older-aged. Males constituted 79 (65.8%) of the participants, whereas females accounted for 41 (34.2%), resulting in a male-to-female ratio of approximately 1.9:1 (Table 1).

**Table 1:** Baseline sociodemographic characteristics of the study participants (N = 120) Data are presented as mean ± SD and median (range) for continuous variables, and as frequency with percentage for categorical variables. Age is expressed in years.

Variable	Category	Value
Age (years)	Mean ± SD	53.5 ± 14.5
	Median (Range)	53 (30–79)
Sex	Male	79 (65.8%)
	Female	41 (34.2%)

Persistent cough was the most common presenting symptom, reported in 28 patients (23.3%). This was followed by dyspnea in 21 patients (17.5%) and prolonged fever in 20 patients (16.7%). Hemoptysis and unexplained weight loss were each observed in 18 patients (15.0%), while chest pain was reported by 15 patients (12.5%).

Radiological evaluation revealed pulmonary nodules as the most frequent finding, identified in 35 patients (29.2%). Non-resolving consolidation was observed in 24 patients (20.0%), and cavitary lesions were noted in 23 patients (19.2%). Lung masses and mediastinal or hilar lymphadenopathy were each present in 19 patients (15.8%) (Table 2).

**Table 2:** Distribution of clinical features and radiological findings among study participants (N = 120) Values are expressed as number (percentage). Clinical features represent presenting symptoms at the time of evaluation. Radiological findings are based on imaging studies performed during diagnostic workup.

Variable	Category	n (%)
Clinical Features	Persistent Cough	28 (23.3)
	Dyspnea	21 (17.5)
	Prolonged Fever	20 (16.7)
	Hemoptysis	18 (15.0)
	Unexplained Weight Loss	18 (15.0)
	Chest Pain	15 (12.5)
Radiological Findings	Pulmonary Nodule	35 (29.2)
	Non-resolving Consolidation	24 (20.0)
	Cavitary Lesion	23 (19.2)
	Lung Mass	19 (15.8)
	Mediastinal/Hilar Lymphadenopathy	19 (15.8)

The mean systolic blood pressure was 106 ± 15.2 mmHg, and the mean diastolic blood pressure was 76.6 ± 9.53 mmHg. The average SpO₂ was 94.3 ± 3.52%, and the mean heart rate was 91.5 ± 18.7 beats per minute. The mean hemoglobin level was 11.9 ± 2.13 g/dL. The mean total leukocyte count was 11,456 ± 3,995/mm³, reflecting a mild elevation in several patients (Table 3).

**Table 3:** Baseline physiological and laboratory parameters of study participants (N = 120) Continuous variables are expressed as mean ± SD, median, and range. SpO₂: Peripheral oxygen saturation

Parameter	Mean ± SD	Median	Range
Systolic Blood Pressure (mmHg)	106 ± 15.2	107	74–134
Diastolic Blood Pressure (mmHg)	76.6 ± 9.53	76	60–94
SpO₂ (%)	94.3 ± 3.52	95	88–99
Heart Rate (bpm)	91.5 ± 18.7	91	61–120
Hemoglobin (g/dL)	11.9 ± 2.13	12.3	8.1–15.3
Total Leukocyte Count (/mm³)	11456 ± 3995	11925	4220–17985

Bronchial biopsy, considered the gold standard, revealed malignancy in 77 patients (64.2%), whereas 43 patients (35.8%) were diagnosed with benign pathology. All four cytological modalities demonstrated a statistically significant association with bronchial biopsy findings (p < 0.001 for all comparisons). Sputum cytology correctly identified 38 of 77 biopsy-proven malignant cases as malignant. However, 21 malignant cases were reported as nonspecific inflammatory, 14 as inadequate, and four as suspicious. Among the 43 biopsy-proven benign cases, 24 were categorized as specific benign and 13 as nonspecific inflammatory, while two cases were incorrectly labeled as malignant. The association between sputum cytology and histopathology was statistically significant (χ² = 63.3, df = 4, p < 0.001). BAL cytology detected 55 of 77 malignant cases as malignant. Nine malignant cases were classified as nonspecific inflammatory, four as suspicious, and nine as inadequate. Among benign cases, 20 were reported as specific benign and 19 as nonspecific inflammatory, with four false-positive malignant reports. The association was statistically significant (χ² = 77.2, df = 4, p < 0.001). TBNA cytology demonstrated the highest concordance with histopathology, correctly identifying 71 of 77 malignant cases. Only one malignant case was reported as nonspecific inflammatory, while five were categorized as suspicious. Among benign cases, 14 were reported as specific benign and 24 as nonspecific inflammatory, with three inadequate samples. The association was highly significant (χ² = 107, df = 4, p < 0.001). Brush cytology identified 66 of 77 malignant cases, with 6 cases categorized as suspicious. Two malignant cases were reported as inadequate, and three as nonspecific inflammatory. Among benign cases, 20 were categorized as specific benign and one as nonspecific inflammatory, while one benign case was incorrectly labelled as malignant. The association was statistically significant (χ² = 103, df = 4, p < 0.001) (Table 4). 

**Table 4:** Comparison of cytological findings across diagnostic modalities stratified by final biopsy result (N = 120) Data are presented as absolute frequencies. The final biopsy result (benign or malignant) served as the reference standard. Chi-square (χ²) test was used to assess the association between cytological findings and biopsy results for each modality. A p-value <0.05 was considered statistically significant. BAL: Bronchoalveolar lavage; TBNA: Transbronchial needle aspiration

Modality	Biopsy Result	Specific Benign	Nonspecific Inflammatory	Malignant	Suspicious	Inadequate	Total	χ²(df)	p-value
Sputum Cytology	Benign (n=43)	24	13	2	0	4	43	63.3( 4)	<0.001
	Malignant (n=77)	0	21	38	4	14	77		
BAL Cytology	Benign (n=43)	20	19	4	0	0	43	77.2 ( 4)	<0.001
	Malignant (n=77)	0	9	55	4	9	77		
TBNA Cytology	Benign (n=43)	14	24	2	0	3	43	107 ( 4)	<0.001
	Malignant (n=77)	0	1	71	5	0	77		
Brush Cytology	Benign (n=43)	20	1	1	0	20	43	103 ( 4)	<0.001
	Malignant (n=77)	0	3	66	6	2	77		

**Figure 1:**
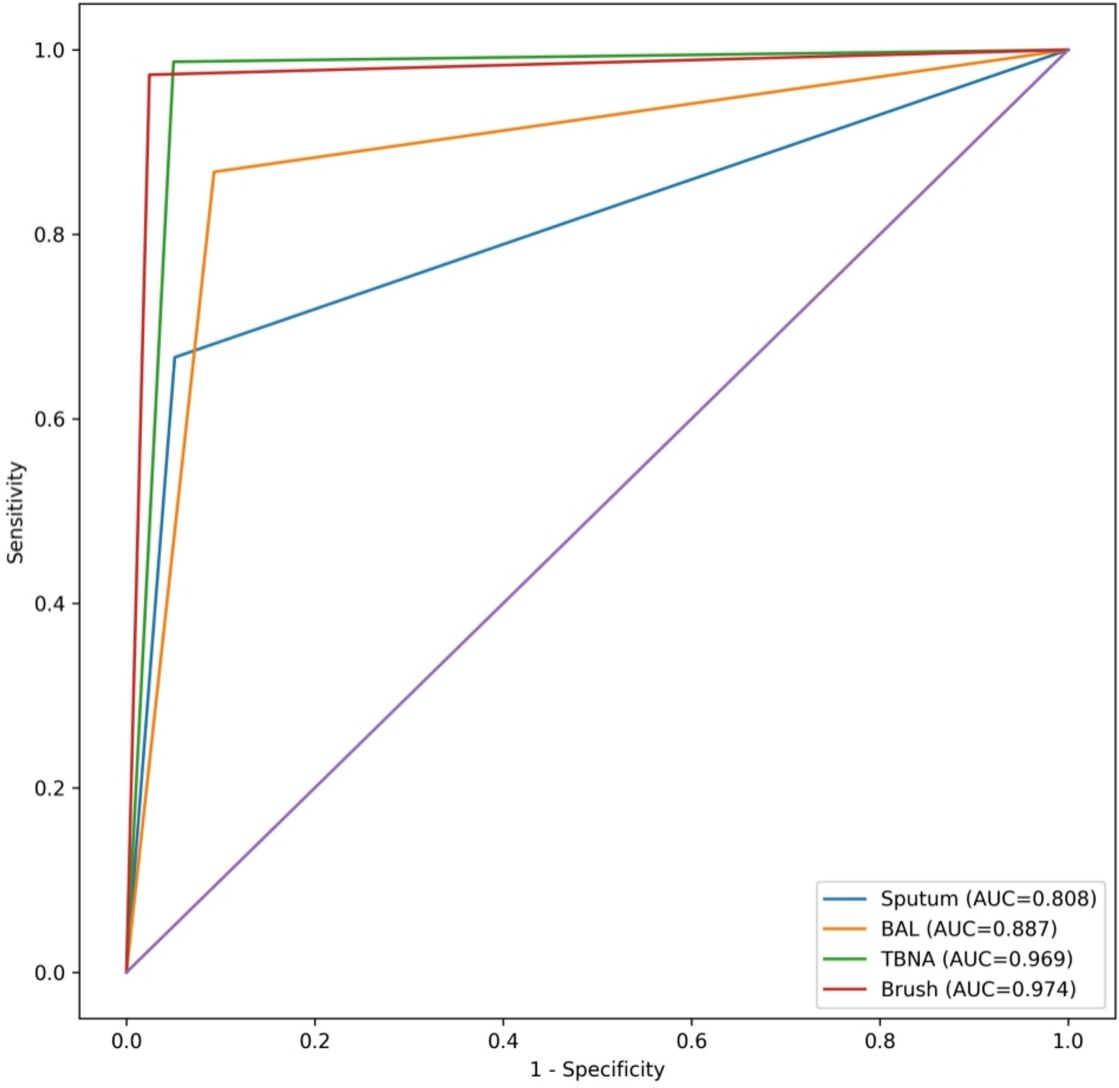
Combined ROC curves comparing diagnostic performance of cytology modalities for detection of malignancy ROC curves demonstrating the diagnostic accuracy of sputum cytology, BAL cytology, TBNA cytology, and bronchial brush cytology in detecting malignant lesions, using final histopathological biopsy as the reference standard. The X-axis represents 1 - specificity (false positive rate), and the Y-axis represents sensitivity (true positive rate). The diagonal indicates the no-discrimination line (AUC = 0.50). The AUC values were 0.808 for sputum cytology, 0.887 for BAL cytology, 0.969 for TBNA cytology, and 0.974 for brush cytology, indicating the highest diagnostic accuracy for brush and TBNA cytology. ROC: Receiver operating characteristic; BAL: Bronchoalveolar lavage; TBNA: Transbronchial needle aspiration; AUC: Area under the curve

Receiver operating characteristic (ROC) curve analysis further evaluated the discriminative performance of each modality. The area under the curve (AUC) for sputum cytology was 0.808, indicating acceptable diagnostic accuracy. BAL cytology demonstrated improved performance with an AUC of 0.887. TBNA cytology exhibited excellent diagnostic accuracy with an AUC of 0.969. Brush cytology showed the highest discriminative ability, with an AUC of 0.974 (Figure 1).

Overall, TBNA and brush cytology demonstrated superior diagnostic performance compared with BAL and sputum cytology for detecting malignant lesions, with bronchial biopsy as the gold standard. For diagnostic accuracy calculations, suspicious cytology results were grouped with malignant cases, while inadequate samples were excluded from performance analysis.

## Discussion

The present study assessed the diagnostic performance of sputum cytology, BAL cytology, TBNA, and brush cytology. These were compared with bronchial biopsy in a mostly middle-aged to elderly group (mean age 53.5 years), with 65.8% male. This demographic profile matches current lung cancer trends. Cytopathology is central for minimally invasive diagnosis and precision oncology. Jain and colleagues have shown that cytology-based approaches are increasingly reliable for lung cancer, especially when integrated with molecular platforms. This supports the assessment of cytological modalities alongside biopsy in this group [9].

Radiologically, pulmonary nodules were the most common finding (29.2%), followed by non-resolving consolidation and cavitary lesions. This predominance of nodular lesions matches meta-analytic evidence, as most diagnostic bronchoscopy studies focus on peripheral pulmonary nodules. Additionally, a lesion size greater than 2 cm and a bronchus sign increase diagnostic yield. Furthermore, the high malignancy rate (64.2%) is similar to reports showing that a higher pretest probability of cancer improves bronchoscopic performance [2].

Sputum cytology in this study showed moderate sensitivity (49.4%) with an AUC of 0.808. While conventional sputum cytology has had limited sensitivity, recent advances, such as automated flow cytometry with machine learning, have achieved sensitivities up to 92% and specificities of 87% for early lung cancer [10]. Additionally, liquid biopsy platforms that use sputum biomarkers are proposed as minimally invasive alternatives to tissue biopsy, particularly for screening and monitoring [11,12]. In comparison, conventional sputum cytology in this group performed lower, highlighting the value of integrating molecular or artificial intelligence (AI) adjuncts.

BAL cytology in this cohort achieved a sensitivity of 71.4% with an AUC of 0.887, demonstrating greater accuracy than sputum cytology. As a diagnostic tool, BAL is valuable for detecting malignancy and inflammatory and interstitial lung diseases, particularly for differentiating granulomatous and eosinophilic disorders [13]. However, its diagnostic performance depends on lesion accessibility and the presence of inflammation. For peripheral lesions, advanced navigation bronchoscopy techniques yield diagnostic rates of about 70.9% and low complication rates (5.6%) [14] - results that are similar to those observed with BAL in this study. Nonetheless, BAL remains less targeted than needle- or device-guided approaches.

TBNA cytology showed excellent diagnostic accuracy in this study, with a sensitivity of 92.2% and an AUC of 0.969, indicating the highest concordance with biopsy. This finding aligns with existing evidence that EBUS-TBNA is a reliable, minimally invasive option for diagnosing and subtyping NSCLC. For example, Karadzovska-Kotevska et al. reported an 83% diagnostic success and high molecular adequacy (epidermal growth factor receptor (EGFR) 69%, anaplastic lymphoma kinase (ALK) 49%, ROS1 36%), reinforcing both the diagnostic and molecular utility of TBNA [15]. Furthermore, decision-analysis modelling shows that bronchoscopy strategies with ROSE are more cost-effective than CT-guided biopsy in selected cases [16]. Thus, these findings suggest that TBNA is a highly accurate and minimally invasive diagnostic modality, particularly for accessible central or nodal lesions. However, its performance is context-dependent, and it should be considered a complementary technique rather than a substitute for biopsy, especially in cases requiring histological architecture or molecular characterization.

Brush cytology had the highest AUC (0.974) and sensitivity of 85.7% in this study. In comparison, reviews of advanced bronchoscopic techniques indicate that new technologies, such as robotic bronchoscopy and shape-sensing systems, achieve diagnostic yields ranging from 81.7% to 84.8%. These methods also offer high navigational success rates (up to 98.7%) and low complication rates (around 3%) [17,18]. Although this study used conventional brush cytology, the similarly high accuracy suggests that well-performed standard techniques remain highly effective in accessible lesions.

Network meta-analyses indicate that CT-guided transthoracic biopsy achieves the highest diagnostic yield (88.9%) but is associated with higher complication rates, while robotic-assisted bronchoscopy attains similar yields (approximately 84.8%) with improved safety profiles [6]. In comparison, the TBNA and brush cytology AUC values observed in this study (0.969 and 0.974, respectively) demonstrate performance comparable to that of these advanced techniques in the clinical context. Additionally, transbronchial lung cryobiopsy has demonstrated diagnostic yields of 76.8-80.7% in interstitial lung disease with acceptable safety profiles, reflecting the broader trend toward minimally invasive bronchoscopic diagnostics as alternatives to surgical biopsy [19,20].

Standardization of diagnostic yield reporting remains essential. Consensus statements emphasize the importance of strict definitions of diagnostic yield and adherence to Standards for Reporting of Diagnostic Accuracy Studies (STARD) guidelines to facilitate reliable comparisons across studies [21]. The use of receiver operating characteristic (ROC) analysis and AUC values in this study enhances interpretability beyond crude yield calculations, aligning with recommendations that favor accuracy-based metrics over simple yield percentages [22]. 

While ROC curve analysis demonstrated excellent diagnostic performance for TBNA and brush cytology, these findings should be interpreted within the context of the selected study population. The absence of lesion stratification and inclusion of predominantly accessible lesions may lead to overestimation of diagnostic accuracy.

Recent advances in molecular pathology and cytology-based biomarker testing further expand the role of cytological samples. Cytology specimens are increasingly regarded as reliable alternatives for molecular testing, providing high-quality DNA extraction and suitability for EGFR, ALK, and ROS1 analysis [23]. Deep learning models applied to cytological images have demonstrated high diagnostic accuracy and AUC values for distinguishing lung cancer subtypes, suggesting that integrating AI into cytology workflows may enhance diagnostic precision [24].

Overall, these findings demonstrate a clear diagnostic hierarchy consistent with the contemporary literature: targeted bronchoscopic techniques, such as TBNA and brush cytology, provide superior diagnostic accuracy compared with BAL and sputum cytology, with bronchial biopsy as the gold standard. Although novel technologies such as robotic bronchoscopy, cryobiopsy, and liquid biopsy continue to advance the field, the results confirm that conventional bronchoscopic cytology, when appropriately applied, remains a highly effective and minimally invasive diagnostic approach for evaluating pulmonary malignancy.

Limitations

This study has several important limitations. First, it was conducted at a single tertiary care center with a relatively modest sample size, which may limit generalizability. Second, verification bias is present because only patients who underwent all diagnostic modalities, including biopsy, were included, potentially inflating diagnostic accuracy by selecting biopsy-accessible lesions. Third, lesion characteristics such as size, anatomical location (central vs peripheral), and bronchus sign were not stratified, a major limitation, as diagnostic yield is highly dependent on these factors. Fourth, procedural variables such as the number of TBNA passes and the absence of ROSE may have influenced diagnostic performance. Fifth, interobserver variability among cytopathologists was not formally assessed. Finally, the lack of follow-up of negative cases may underestimate false-negative rates and overestimate specificity.

A critical consideration in interpreting these findings is that all included patients underwent successful bronchoscopy with biopsy, indicating that lesions were accessible for tissue sampling. This introduces verification bias, as diagnostically challenging or inaccessible lesions were not represented. Consequently, the observed high concordance between cytology and biopsy may reflect sampling from biopsy-friendly lesions rather than independent diagnostic equivalence. The lack of lesion stratification further limits interpretation, as diagnostic yield varies significantly between central and peripheral lesions.

Future scope of the research

Future research should include multi-center, large-scale studies to validate these findings across diverse populations and healthcare settings. Stratified analyses based on lesion size, bronchus sign, and radiological characteristics are needed to refine diagnostic algorithms. Systematic evaluation of ROSE, liquid-based cytology, and molecular testing within routine workflows is recommended to accelerate time to definitive diagnosis and facilitate earlier initiation of therapy. Comparative studies of robotic bronchoscopy, cryobiopsy, and AI-assisted cytology are needed to define optimal minimally invasive strategies for earlier stage detection and improved five-year survival. Cost-effectiveness analyses are essential in resource-limited settings to maximize patient benefit. Longitudinal follow-up studies assessing clinical outcomes and survival correlations would provide stronger evidence for adopting cytology-driven diagnostic pathways in pulmonary oncology and support improved patient-centered outcomes.

## Conclusions

Bronchoscopy-guided cytological techniques, particularly TBNA and brush cytology, demonstrate high diagnostic accuracy and strong agreement with bronchial biopsy in detecting malignant pulmonary lesions. However, these findings primarily reflect performance in biopsy-accessible lesions. Therefore, cytological techniques should be considered complementary diagnostic tools rather than replacements for biopsy. Their optimal use lies in enhancing diagnostic yield within multimodal evaluation strategies, especially in resource-limited settings or when biopsy is not feasible.
